# Does the autism phenotype differ when selecting groups by neurodevelopmental versus genetic diagnosis? An observational study comparing autism and sex chromosome trisomy

**DOI:** 10.12688/f1000research.121878.1

**Published:** 2022-05-25

**Authors:** Alexander C Wilson, Dorothy V M Bishop

**Affiliations:** 1 Department of Experimental Psychology, University of Oxford, Oxford, UK

**Keywords:** Autism, autism phenotype, sex chromosome trisomy, diagnostic criteria, language

## Abstract

**Background:** Autism is diagnosed on the basis of social and non-social behavioural features that are assumed to cluster together, and assumed to be distinct from other aspects of development, such as language ability. It is unclear, however, if these assumptions are valid. This study presents a novel approach to answering this question by investigating whether correlations between autism features are similar for groups selected on behavioural versus genetic diagnosis.

**Methods:** The autism phenotype was assessed by diagnostic interview in young people aged 7 to 14 diagnosed with autism ( *N*=61) or sex chromosome trisomy (SCT; *N*=49). Data were analysed by confirmatory factor analysis and MANOVA.

**Results:** Autism features showed a similar factor structure and were distinct from language ability in both groups. However, the SCT group was more likely to show clinically-significant difficulties in just some aspects of autism and a lower level of non-social autism features for their social-communication disabilities.

**Conclusions:** We suggest the group differences emerged because autism diagnostic criteria do not map exactly on the autism phenotype as it manifests “naturally”. Conventional diagnostic criteria for autism miss those with uneven profiles of difficulty and those with relatively low levels of restricted and repetitive behaviours and interests.

## Introduction

Autism, like other neurodevelopmental disabilities, is diagnosed on the basis of a cluster of behavioural features that are assumed to group together as a single “entity” underpinned by specific neurocognitive mechanisms. In the case of autism, these features cluster across two domains: (1) social interaction and communication, and (2) non-social features including preference for routine, highly focused interests, repetitive behaviours and sensory processing differences ( American Psychiatric Association, 2013). While autistic-like traits vary continuously through the population, it is assumed that there is a threshold where these traits become “clinically significant” and a diagnosis is deemed useful. Autism is also defined by what it is not: although autistic individuals may also have disabilities affecting core language (speech, syntax, vocabulary, etc.), these are not a cardinal feature of autism and where they occur are assumed to be separate from autism, forming a so-called “co-occurring” disability. Characterising neurodevelopmental disability in this way raises longstanding questions about diagnostic validity: specifically, about how we ought to lump and split neurodevelopmental features across different diagnostic categories. In the case of autism, does it reflect a natural category where social and non-social features go together? And likewise, is this category ultimately distinct from language ability?

### Do the social and non-social features of autism naturally group together?

**Figure 1.**
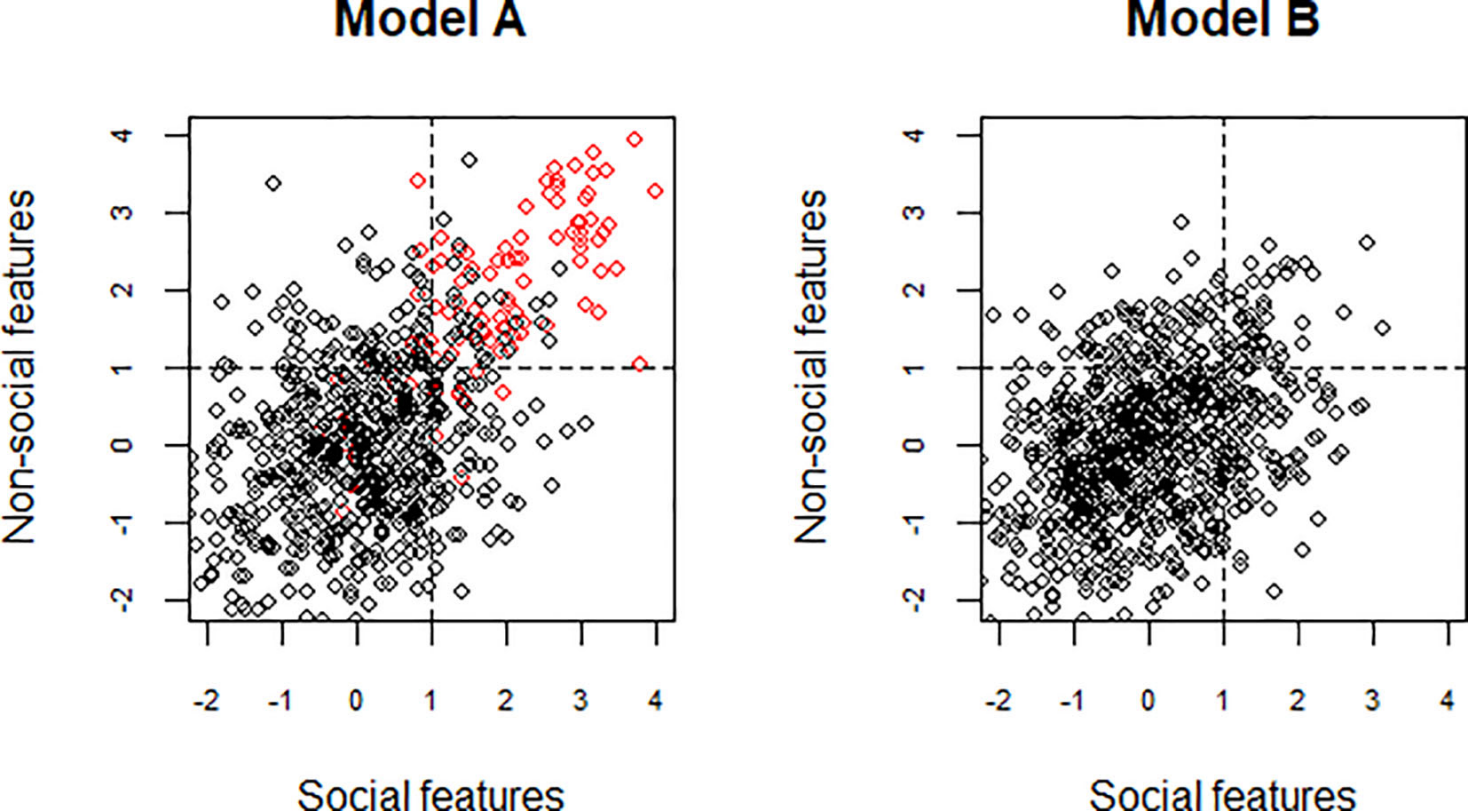
Plot showing two possibilities for the relationship between social and non-social features of autism in the population. In model A, there is a distinctive autism subgroup in addition to the general population. In this autism subgroup (shown as red circles), social and non-social features closely co-occur. By contrast, among non-autistic people (shown as black circles), social and non-social features are only weakly associated. In model B, there is no distinctive autism subgroup, but rather a wide variation in the distribution of social and non-social features across the population.

When autism is defined in terms of a combination of social and non-social features, we implicitly adopt a model such as that shown as Model A in Figure 1. Here, the black circles denote non-autistic individuals, for whom social and non-social features are only weakly associated, and the red circles denote those with autism, for whom both social and non-social features co-occur. A positive score on each scale denotes impairment. The dotted lines denote possible cut-offs that could be used diagnostically, and which would do a good job at identifying the subset of red dots as those in the top right quadrant. Another possibility, however, is that reality corresponds to model B, in which there is no distinctive autism subgroup, but rather a wide variation in the distribution of social and non-social features. Even though there is no distinctive autism subset in model B, we can still place cut-offs to identify those in the top right quadrant. Note that in model B, but not in model A, there are numerous people who score above threshold on either social or non-social features, but not both.

There are a couple of sources of information which are consistent with model B rather than model A. First, some individuals show an uneven profile of difficulties, with social communication problems but a relative absence of other autistic features. This includes individuals with pragmatic language impairment ( Gibson *et al.*, 2013) and pervasive developmental disorder – not otherwise specified ( Mandy *et al.*, 2011). The dissociation is not entirely clear-cut, however, because such individuals may show some evidence of elevated but subthreshold autism features in the non-social domain too. A more secure source of information is from factor-analytic studies, and these provide evidence for two dimensions of autism features, rather than a single autism “entity”. Empirical studies consistently find separable social and non-social factors when assessing questionnaire and interview-based data on autistic individuals (see Shuster *et al.*, 2014 for a review). Clinical assessments indicate a moderate correlation between social and non-social features of autism in autistic groups (e.g. 0.44 in over 600 autistic children, Mandy *et al.*, 2014). In contrast, in general population groups, correlations tend to be low, although these are based on non-clinical measures relating to autistic-like traits rather than autism features per se (e.g. Svedholm-Häkkinen *et al.*, 2018). In addition, genetically-informative studies indicate that these domains are highly heritable, but that genetic influences on the domains are relatively independent ( Ronald *et al.*, 2006). While these results consistently come to the same conclusion about the separability of autistic features into social and non-social domains, there are possible issues to consider regarding the samples and measures.

With respect to samples, it may not be optimal to rely either on autistic or general population groups. Autistic individuals will necessarily show elevated difficulties in both domains as this is required for diagnosis, and so there will effectively be a selection bias towards those with both social and non-social impairments. On the other hand, in general population groups, there may be an issue around generalisability and validity of measures. It has been found that genetic influence on autistic-like traits is shared across general population and autistic groups (e.g. Robinson *et al.*, 2016), and so we can be confident that it is valid to study autistic-like traits across the general population. However, it might be the case that autistic-like traits are not *directly* equivalent to the clinical features of autism. It is clear that the questionnaires used for screening and measuring autistic-like traits in the general population (such as the Autism-Spectrum Quotient; Baron-Cohen *et al.*, 2001) do not necessarily map well on to clinical assessments used to support an autism diagnosis (such as the Autism Diagnostic Observation Schedule; Lord *et al.*, 2012). For instance, an epidemiological study of autism prevalence found a correlation of just 0.24 between these measures in a sample of over 600 adults oversampled for possible autism ( Brugha *et al.*, 2012). This underscores the possible limitations of relying on general population samples for understanding the relationship between the different domains of autism features, as studies of such samples will not contain individuals with clinically-significant difficulties, and are likely to rely on non-clinical questionnaire measures. Pulling together the limitations explored here, a more optimal approach might be to measure autism features in the social and non-social domains using a clinical assessment in a sample that has elevated likelihood of developmental disability but has not been selected on the basis of their neurodevelopmental presentation. Instead of neurodevelopmental presentation, we can select on the basis of aetiology. We will present results from children with a sex chromosome trisomy to illustrate this approach.

### Is “autism” distinct from language ability?

Language problems introduce a further layer of complexity into the diagnosis of autism. There is a greater risk of language difficulties among autistic people compared to the general population ( Kwok *et al.*, 2015), and a significant proportion of autistic individuals (perhaps 30%) are minimally verbal ( Tager-Flusberg & Kasari, 2013). Language difficulties may accentuate autism features, given that verbal ability impacts on scores on autism diagnostic assessments ( Hus *et al.*, 2014), and such difficulties predict a range of later outcomes for autistic people ( Magiati *et al.*, 2014). As language functioning is so heterogeneous and relevant to outcome in autism, DSM-5 suggests making use of a specifier in autism diagnosis indicating whether language impairment is present or not ( American Psychiatric Association, 2013).

When considering language problems, we need to distinguish between different aspects. Individuals can have problems with understanding or producing grammatical sentences or learning new vocabulary, but these core language difficulties can be dissociated from problems with language use (pragmatics), which are a cardinal feature of autism. Some autistic people may present with social communication problems in the absence of language difficulties or indeed with advanced language development ( Baird & Norbury, 2016; Lam & Yeung, 2012). This dissociation between social communication and language is also captured in the theoretical models, which typically attribute the social difficulties in autism to cognitive constructs, such as “theory of mind”, while under-emphasising language (e.g. Baron-Cohen, 2000). Nevertheless, empirical evidence indicates that pragmatic difficulties in autism typically co-occur with core language difficulties, as reviewed by Andres-Roqueta and Katsos (2017) and Matthews *et al.* (2018). Furthermore, children who are selected on the basis of having language impairments tend to have elevated social communication difficulties ( Leyfer *et al.*, 2008; Norbury *et al.*, 2004). This raises the possibility that dissociations between pragmatics and core language skills may have been exaggerated, with core language problems going under-recognised in autistic individuals because the pragmatic difficulties are more striking and obvious.

### A novel approach to examining the relationships between different neurodevelopmental features: comparing genetically and behaviourally defined samples

A sample selected on the basis of a clinical diagnosis will necessarily show elevated levels of the traits relevant to the diagnosis – i.e. in the case of autism, social communication difficulties and restrictive and repetitive behaviours and interests (RRBIs). This may make social communication difficulties and RRBIs appear more connected than they really are and may also underplay relationships with other neurodevelopmental features. A reverse situation is also possible where selecting a sample with clinically-significant difficulties, i.e. with a diagnosis of autism, may deflate relationships between neurodevelopmental domains due to restricted variance. To summarise, it is possible that an autistic sample will lead us to see a biased picture of the relationships between different neurodevelopmental domains. To assess whether this is the case, we can compare (1) a sample that meets behavioural criteria for autism to (2) a sample selected on aetiological rather than behavioural grounds – in the case of this study, individuals with a genetic variation linked to neurodevelopmental disability. Effectively, in the first sample, we have a group where biases of the diagnostic system may be causing features of neurodevelopmental disability to cluster or fail to cluster; whereas, in the second sample, we have a genetically-defined group where we might see more accurately how nature lumps and splits features of neurodevelopmental disability. This second sample might not represent how such features cluster continuously across the population, but it does give us confidence that there might be neurocognitive reasons rooted in genetics that lead to particular patterns of neurodevelopmental traits. Having selected autistic and genetically-defined samples, we then compare the relationships between different neurodevelopmental traits across the groups. Where we see no discrepancies, we can infer that the diagnostic system maps on appropriately to the structure of neurodevelopmental disability as it manifests “naturally”. If there are differences, there may be sources of bias that are worth investigating further.

For the genetically-defined sample, the present study investigated a group of young people with a sex chromosome trisomy. Sex chromosome trisomy occurs where an individual has an additional X or Y chromosome; karyotypes include 47,XXX (Trisomy X), 47,XXY (Klinefelter’s Syndrome) and 47,XYY (Jacob’s Syndrome). These are common genetic variations occurring in about one of 650 to 1000 same sex births depending on the karyotype ( Morris *et al.*, 2008). All three karyotypes are associated with increased probability of neurodevelopmental disability, including delayed early milestones, language and literacy difficulties, executive dysfunction, autism, ADHD and anxiety (see van Rijn, 2019 for a review). The phenotype is variable, and many individuals will not show indications of neurodevelopmental disability, meaning that the majority of individuals with an SCT are likely to go undiagnosed ( Abramsky & Chapple, 1997). Those who are diagnosed typically fall into two subgroups: (1) individuals identified incidentally on the basis of routine prenatal screening and (2) individuals identified postnatally following clinical investigations due to medical or behavioural concerns, who will therefore typically show a more severe phenotype than those diagnosed prenatally ( Wilson *et al.*, 2019). These issues around clinical ascertainment mean that individuals with a milder phenotype will typically be underrepresented in research samples, whereas clinically referred individuals will be overrepresented. The result is that mean scores for neurodevelopmental disability will be inflated compared to the true population level. This is highly problematic for research that aims to identify the “typical” phenotype in SCTs; however, for the purpose of the present study, this is less of an issue, as we are interested not in mean scores but in variances and covariances – how variability in one domain relates to variability in another domain of neurodevelopmental disability.

In the present study, we compared the structure of neurodevelopmental disability across samples defined by behavioural vs genetic diagnoses. We hypothesised that autism features would cluster differently across groups, and that language ability would show different relationships with autism features across groups. In terms of the clustering of autism features, we predicted that young people in the SCT group might be more likely to show elevated features in just one domain (e.g. just social communication), leading to greater dissociations in this group. As for language ability, we predicted that the link between language and social communication may be attenuated in the autistic group if the presence of an autism diagnosis selects for individuals whose communication problems are more attributable to social-cognitive rather than linguistic factors.

These issues surrounding clustering of neurodevelopmental traits are of theoretical interest, but there is also a practical issue here worth exploring: how does an individual’s specific clustering of difficulties, as represented by their diagnostic label, influence the clinical and educational support given? In particular, is support sometimes more dependent on the label than the actual functional difficulties the individual is experiencing? We therefore asked whether young people with an autism diagnosis and young people with an SCT, when controlling for level of difficulties, received different levels of special educational needs (SEN) support.

## Methods

The study was granted ethical clearance in November 2018 by the Medical Science Interdivisional Research Ethics Committee [R59912] at the University of Oxford.

### Participants

We recruited families of young people aged 7;0 to 14;11 years who were diagnosed with a sex chromosome trisomy and/or autism spectrum condition. Families were recruited through social media and support groups, including Autistica, the National Autistic Society, Unique - Rare Chromosome Disorder Support Group, the Klinefelter Syndrome Association and The Association for X and Y Chromosome Variations. Exclusion criteria included: (1) severe and uncorrected sensory impairment, (2) history of neurological illness or brain injury, (3) nonverbal or word/phrase-level speech, and (4) English spoken as an additional language. To screen for limited language, families were asked “We would like to know whether your child can speak in sentences such as ‘I didn’t go the park because it rained’ and ‘I think the film was really good’. Your child may make mistakes when using sentences, but can they at least sometimes use sentences like these?” To be included in the study, families needed to say ‘yes’ or ‘maybe’ in response to this question.

110 families entered the study; 49 were in the SCT group and 61 in the autistic group. In the SCT group, all families reported that the SCT diagnosis followed chromosomal microarray testing. Families in this group reported the following additional diagnoses: autism ( *n* = 10), ADHD ( *n* = 10), specific language impairment/developmental language disorder ( *n* = 20), dyslexia ( *n* = 8), dyspraxia/developmental coordination disorder ( *n* = 7), and anxiety ( *n* = 7). Most families were recruited specifically for this study, though seven had participated in our previous study ( Wilson *et al.*, 2019). In the autistic group, all families reported that the autism diagnosis followed a clinical interview with the family and behavioural observation of the child, mostly within multidisciplinary services including psychologists, paediatric doctors and speech and language therapists. Families in the autistic group reported the following additional diagnoses: ADHD ( *n* = 13), specific language impairment/developmental language disorder ( *n* = 6), dyslexia ( *n* = 6), dyspraxia/developmental coordination disorder ( *n* = 7), and anxiety ( *n* = 12).

A control group was not recruited for this study, although we assessed how performance of our sample on the language battery compared to a normative group of 390 young people recruited from mainstream school ( Wilson & Bishop, 2022a). We ran a nonparametric MANOVA with the five language tests as dependent variables and autism diagnosis, trisomy diagnosis and age as predictors; both diagnosis variables were included as categoric variables coded as 0 or 1, allowing for the few young people with dual diagnoses. Controlling for age, MANOVA indicated that an autism diagnosis was not related to performance across the five language tests, *p* = .324, whereas an SCT diagnosis was linked to underperformance, *p* < .001.

### Procedure

Caregivers completed an online survey in which they were asked about their child’s developmental history and completed two questionnaires, the Pediatric Symptom Checklist-17 ( Gardner *et al.*, 1999), and Children’s Communication Checklist ( Bishop, 1998). Following the survey, we carried out a telephone-based assessment for autism features using the short version of the Developmental, Diagnostic and Dimensional Interview (3Di-sv; Santosh *et al.*, 2009). In the final stage, young people were asked to complete an online battery of newly devised language tasks and a measure of nonverbal reasoning, the Animal Matrices. Families were offered several formats for this assessment session: with the first author at home, school or the University of Oxford, or in the family’s own time without a researcher present. All online aspects of the study were supported by Gorilla ( https://gorilla.sc/). Consent from parents/guardians was sought at the beginning of each stage: on an online form at the beginning of the survey, using an oral consent script in the interview, and an online/paper form prior to the cognitive/language assessment. Young people gave assent to take part in the cognitive/language assessment, and ongoing assent was checked throughout all in-person sessions.

### Measures

***Children’s Communication Checklist (CCC;*** ***Bishop, 1998*** ***)***

In this 70-item questionnaire about communication difficulties, caregivers indicate whether the statement “does not apply”, “somewhat applies” or “definitely applies” to their child (which are assigned scores of 0, 1 or 2 respectively). There is also a “can’t judge” option. The items are grouped into nine subscales covering structural aspects of language (speech and syntax), social aspects of language use, i.e. pragmatics (inappropriate initiation, coherence, stereotyped conversation, use of context, and rapport), and autistic features (social relationships and interests). Although an updated standardised version of this measure exists, this earlier form is integrated with the 3Di-sv, so was used in this study. As questionnaire responses were processed slightly differently to Bishop (1998), scoring is detailed below. Positively-worded items were reverse coded, and items were summed into composite scores, prorating for up to 20% of items where there were missing data. For the present study, only the structural language composite was analysed. Cronbach’s alpha for this composite was.93 [.91, .95].

***Pediatric Symptom Checklist-17 (PSC-17;*** ***Gardner et al., 1999*** ***)***

This is a screening questionnaire devised for use in primary healthcare for identifying children at risk of psychosocial problems. It consists of 17 items that caregivers report as applying to their child “never”, “sometimes” or “often” (scored as 0, 1 or 2 respectively). There are subscales for attentional (5 items), internalising (5 items) and externalising problems (7 items), with cut-offs of 7, 5 and 7 indicating clinically-significant difficulties in these areas. The cut-off on the full scale is 15 and translates to a t-score of 62. The cut-offs have been validated in a study of over 80,000 US families not in the care of a developmental paediatrician who were accessing primary healthcare; positive screening rates were 9 to 12% across the subscales and total scale. Where more than three items are left unanswered, the questionnaire is invalid; with three or fewer unanswered items, total scores are computed as normal, ignoring the missing items. Missing items invalidated total scores on the individual subscales.

***Special educational needs (SEN) provision***

During the survey, families were asked to describe any support their child received for their education, and also whether their child attended special school and/or had an Education, Health and Care Plan or similar statement of additional needs. Responses were coded on a 4-point scale: 0 = no or only occasional additional support; 1 = weekly low-intensity support in mainstream school, e.g. some one-to-one or additional group work; 2 = high-intensity support in mainstream school, e.g. has a dedicated teaching assistant for most lessons; 3 = attends special school. Home-schooling was coded as NA.

***Short version of the Developmental, Diagnostic and Dimensional Interview (3Di-sv;*** ***Santosh et al., 2009*** ***)***

This is a 53-question informant interview for assessing and diagnosing autism, which was administered over the phone in approximately 30 to 40 minutes for each child. The questions are arranged in subscales that contribute to total scores for each of the dimensions of the autistic triad: social interaction, communication, and restricted and repetitive behaviour and interests (RRBIs). The three dimensions have clinically significant cut-offs at 10, 8 and 3. Alongside these continuous variables, an algorithm based on DSM-IV criteria outputs diagnostic classification as autistic or non-autistic. The original validation study showed 93% agreement in case-ness for autism with the Autism Diagnostic Interview-Revised (ADI-R; Lord *et al.*, 1994).

***Receptive vocabulary***

Participants hear a sequence of words and for each word, they are presented with four pictures on the screen. They are asked to “chose which picture goes best with the word”. Participants are given a point for each correct answer for a total score out of 39. Cronbach’s alpha [95% CI] in this sample was.78 [.71, .84]. This measure showed a correlation of.69 with a standardised vocabulary test ( Wilson & Bishop, 2022a).

***Receptive grammar***

Participants listen to sentences and decide if they are grammatical. There are four randomly ordered sentences, 23 items that do not follow typical syntax or use incorrect word forms (e.g. incorrect tenses) and 23 items that follow typical English grammar. Participants are given a point for each correct answer for a total score out of 50. Cronbach’s alpha [95% CI] in this sample was.87 [.83, .91].

***Implicature comprehension test***

Participants watch a series of cartoon videos, with two characters producing a short utterance one after the other. Together the utterances form a conversational adjacency pair; in most cases, this is a question and answer. After this dialogue, participants hear a comprehension question, and they give a yes-no-don’t know response by clicking buttons on the screen. For 33 items, participants need to process implied meaning to answer the question, as the second character provides an indirect response to the first character. Participants are given a point for each correct answer for a total score out of 33. Cronbach’s alpha [95% CI] in this sample was.79 [.73, .84].

***Children’s test of local textual inference***

Participants hear two brief sections of a short story (about 90 words per part). After each section, they hear ten questions and four possible answers for each one. Participants click the correct option on the screen. As well as auditory presentation of all materials, everything is shown in text-based form on the screen. Participants are informed at the start that the short story sections will remain on the screen while they are answering questions about that section. Participants need to make inferences based on the short story to answer the questions. Participants are given a point for each correct answer for a total score out of 20. Cronbach’s alpha [95% CI] in this sample was.78 [.71, .84].

***Social overtures***

Participants hear a series of utterances spoken by a character to a conversational partner. Eleven are social overtures that attempt to engage the partner in a conversation (e.g. “I can’t believe what happened today.”) and twelve are not conversational bids (e.g. “I’m going to have a shower now.”). For each utterance, participants are asked whether the speaker wants a conversation or not, and to indicate their answer by clicking yes-no buttons. Participants are given a point for each correct answer for a maximum total of 23. Cronbach’s alpha [95% CI] in this sample was.84 [.79, .88].

***Nonverbal reasoning (animal matrices)***

In this non-verbal reasoning task, participants see a series of 2×2 matrices presented on the computer screen. In three of the boxes of each matrix, there are cartoon pictures of animals, and the fourth box is empty. The animals in the three boxes vary in systematic ways, and participants deduce which of five options fits in the empty box. Participants are given a point for each correct answer for a maximum total of 16. Cronbach’s alpha [95% CI] in this sample was.83 [.78, .88]. This measure showed a correlation of.70 with a standardised nonverbal reasoning test ( Wilson & Bishop, 2022a).

### Data analysis

All analysis was implemented using R software ( R Core Team, 2020). Plots were generated using R packages ggplot2 ( Wickham, 2016) and ggpubr ( Kassambara, 2020). Data and scripts are available on the Open Science Framework: https://osf.io/qan7r/.

We considered how the autism phenotype manifested in the two groups using three methods. First, we carried out a categoric analysis in which we subdivided individuals based on whether they met autism criteria on one, two or all three dimensions of the 3Di-sv assessment. We tested whether there was a significant difference in the proportion of young people meeting criteria on just one or two dimensions between the SCT and autistic groups using a chi-square test. For our second analysis, we assessed whether autism features measured as continuous variables patterned differently across groups. This involved testing for factorial invariance of a latent “autism features” factor using multi-group confirmatory factor analysis implemented by R package lavaan with robust estimation ( Rosseel, 2012). The three continuous variables produced in the 3Di-sv assessment for social interaction, communication and RRBIs were set to load on the factor. This structure was run separately in the two groups as we incrementally fixed the loadings, intercepts and residuals of the indicators to be the same across groups. Each increasingly constrained model was compared to the previous one using a chi-square test with Satorra-Bentler correction to test whether model fit was significantly affected. Where model fit declined, this would indicate a difference in how the autism phenotype manifested across groups. Finally, in our third analysis, we looked in more detail at the 3Di-sv, specifically at the subscale scores that contribute to the three autism dimensions. We tested whether some subscales contributed disproportionately to the autism phenotype when comparing groups. For each subscale, we used logistic regression to determine the odds that the groups would score differently on the subscale when controlling for total scores on the dimension to which the subscale belonged.

**Figure 2.**
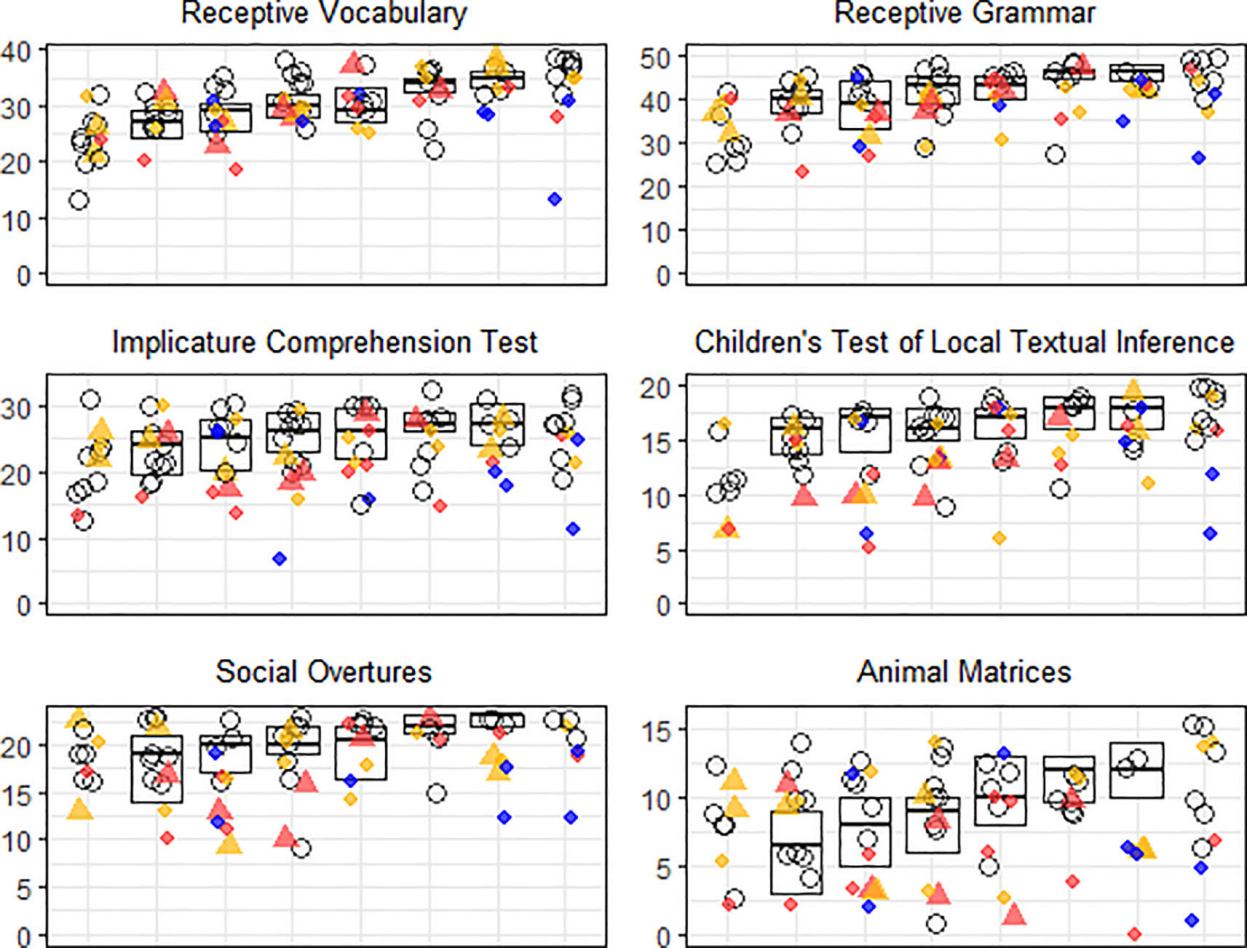
Raw data on the language test battery arranged by school year group along the x-axis, with year 2 (6 to 7-year-olds) to the far left and year 9 (13 to 14-year-olds) to the far right. The box-plots show first, second and third quartiles in the normative sample of children in mainstream school. Unfilled circles show young people in the autistic group, whereas coloured shapes show young people with a sex chromosome trisomy (SCT). The triangles show prenatally diagnosed SCTs and diamonds show postnatally diagnosed SCTs. Trisomy type is coded by colour: red for 47,XXX, gold for 47,XXY, and blue for 47,XYY.

Next, we assessed how language ability related to the autism phenotype across groups. Language ability was measured through parent-report on the CCC as well as performance on the language battery. See Figure 2 for raw data on the language battery. Among children who sat the battery, 3% of tests were not completed and these missing data were handled with multiple imputation implemented by R package mice ( van Buuren & Groothuis-Oudshoorn, 2011). Then a factor score was extracted from the test battery using R package psych ( Revelle, 2019). Analysis of eigenvalues indicated that one factor accounted for performance on the battery, and this explained 61% of variance across the five tests. We removed the effect of age on the language factor score using linear regression. To establish the influence of language on autism features, we used non-parametric MANOVA implemented by R package RVAideMemoire ( Hervé, 2020). The dependent variables were the 3Di-sv dimensions (Social interaction, Communication and RRBIs), and the independent variables were language ability, group and the interaction. This analysis was run twice with language ability operationalised as the factor score extracted from the test battery first, and then parent-reported language difficulties on the CCC structural language scale.

Finally, we tested whether the groups differed in the amount of special educational needs (SEN) provision they received, controlling for their level of difficulties. A two-stage multiple regression was run. In the first stage, we entered age, language test performance, 3Di-sv “autism features” and general psychosocial difficulties measured by the PSC-17, as control variables. (The 3Di-sv variable was extracted from the factor model described above. As the RRBI intercept varied between groups, this was allowed to differ when extracting factor scores). In the second stage of the multiple regression, SCT diagnosis was included as a further predictor.

## Results

**Figure 3.**
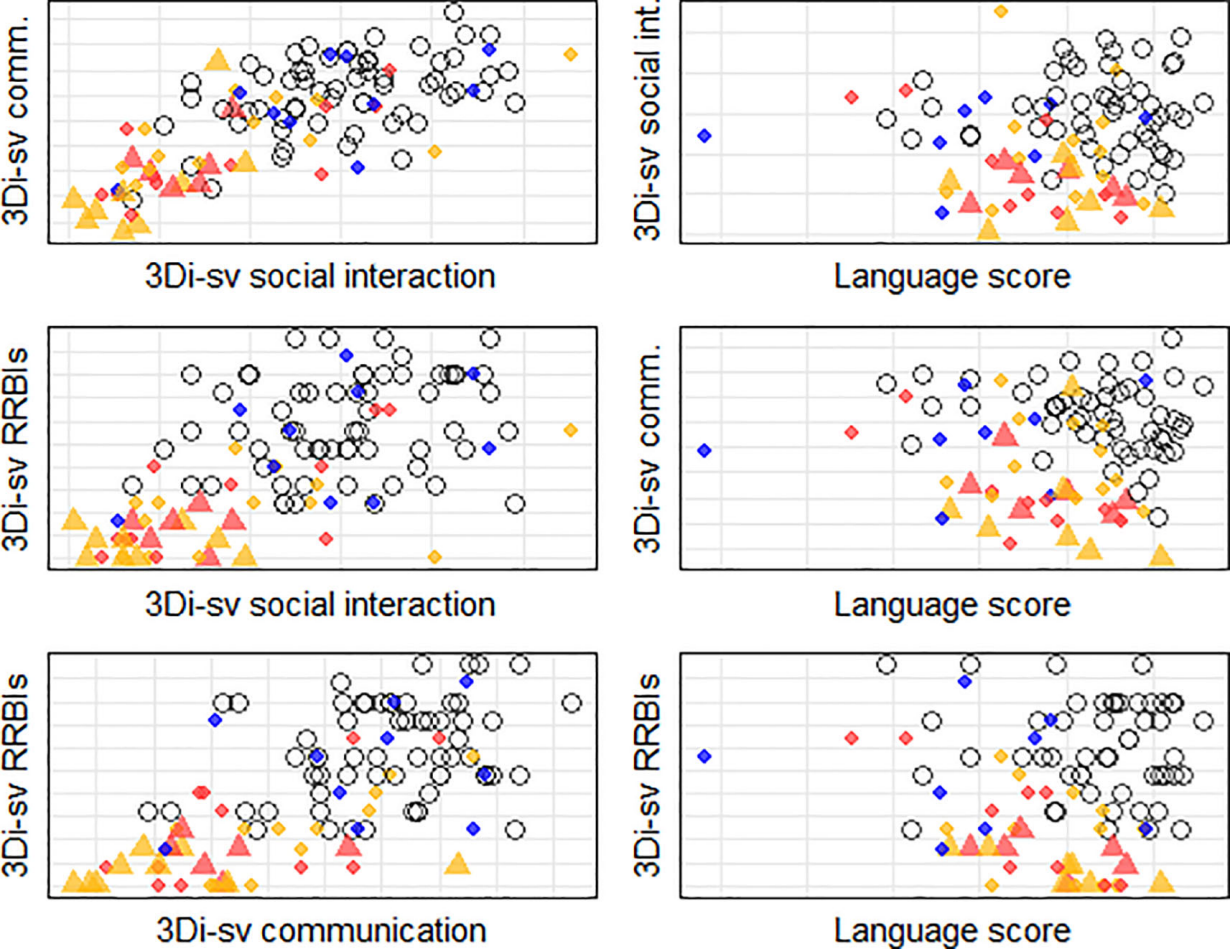
Plots showing scores on the three dimensions (social interaction, communication, and repetitive and restrictive behaviours and interests [RRBIs]) of the 3Di-sv autism assessment and language factor scores extracted from the test battery against one another. Unfilled circles show young people in the autistic group, whereas coloured shapes show young people with a sex chromosome trisomy (SCT). The triangles show prenatally diagnosed SCTs and diamonds show postnatally diagnosed SCTs. Trisomy type is coded by colour: red for 47,XXX, gold for 47,XXY, and blue for 47,XYY.

Table 1 shows a breakdown of the sample and descriptive statistics for all measures used in the following analyses. In the table, we break down mean scores in the SCT group by timing of diagnosis, as postnatally diagnosed individuals tend to show a more marked phenotype. However, for the analyses that follow, the SCT group is combined, as we are interested in clustering of features and variability rather than mean scores. As subdividing the SCT group by trisomy type (i.e. 47,XXX, 47,XXY and 47,XYY) was not viable due to small numbers, we leave aside the issue of trisomy type in the analysis, but raw data shown in Figures 2 and 3 are coded by trisomy type and timing of diagnosis for the reader’s interest.

**Table 1.** Sample characteristics. On the psychometric measures, higher scores indicate greater difficulties, except for the language factor score and nonverbal reasoning where higher scores indicate higher performance.

	Prenatal SCT group	Postnatal SCT group	Autistic group
*N* completing 3Di-sv	15	34	61
*N* completing language battery	13	29	51
Age (years;months)	*M* = 9;11 *SD* = 2;1	*M* = 10;10 *SD* = 2;4	*M* = 10;5 *SD* = 2;3
Parent-reported karyotype	47,XXX ( *n* = 6) 47,XXY ( *n* = 9)	47,XXX ( *n* = 11) 47,XXY ( *n* = 13) 47,XYY ( *n* = 10)	46,XX ( *n* = 17) 46,XY ( *n* = 44)
Autism phenotype			
3Di-sv social interaction	4.96 (2.99)	10.89 (6.79)	15.08 (4.97)
3Di-sv communication	6.76 (4.41)	11.31 (4.64)	15.25 (3.79)
3Di-sv RRBIs	1.13 (0.99)	3.94 (3.18)	7.31 (2.66)
Language and general ability			
CCC structural language	7.20 (6.89)	11.35 (7.45)	7.02 (5.77)
Language factor score	-0.14 (0.68)	-0.53 (1.11)	0.34 (0.87)
Nonverbal reasoning	-0.39 (0.87)	-0.49 (1.09)	0.40 (0.80)
General psychosocial impairment			
PSC attention	5.13 (1.68)	6.06 (2.17)	6.85 (1.86)
PSC internalising	4.53 (2.92)	5.59 (2.39)	5.73 (2.71)
PSC externalising	4.73 (2.49)	4.84 (3.18)	5.69 (2.98)
PSC total score	14.40 (5.05)	16.52 (5.57)	18.20 (5.10)
Special educational needs support			
None	3	6	10
Low-intensity	11	18	24
High-intensity	0	7	11
Special school	1	2	11

*Note.* Language factor scores and nonverbal reasoning scores have been age-adjusted and z-transformed to allow comparison across groups.

### Autism phenotype across SCT and autistic samples

Figure 3 shows raw data on the three dimensions of the 3Di-sv. We assessed the relative proportions of young people meeting autism criteria across these dimensions. As shown in Table 2, young people with an SCT were more likely to show uneven profiles, with clinically significant difficulties in just some aspects of the autism phenotype. 19 of 49 (39%) of the SCT sample met clinical criteria on just one or two of the autism dimensions of the 3di-sv, compared to 9 of 61 (15%) of the autistic sample; this difference was significant, Χ ^2^ (1) = 7.05, *p* = .008.

**Table 2.** Number of children above-threshold on the 3Di-sv criteria for autism. Table shows number of children (percentage of total).

	Prenatal SCT group			Postnatal SCT group			Autistic group
Karyotype	*47,XXX*	*47,XXY*	*47,XYY*	*47,XXX*	*47,XXY*	*47,XYY*	
*N* children with 3Di-sv data	*6*	*9*	*0*	*11*	*13*	*10*	*61*
Over threshold in one dimension							
Social interaction	0 (0)	0 (0)	0 (0)	1 (9)	0 (0)	0 (0)	0 (0)
Communication	3 (50)	2 (22)	0 (0)	0 (0)	2 (15)	0 (0)	0 (0)
RRBIs	1 (17)	0 (0)	0 (0)	1 (9)	1 (8)	0 (0)	2 (3)
Over threshold in two dimensions							
Social interaction AND communication	0 (0)	0 (0)	0 (0)	1 (9)	1 (8)	0 (0)	0 (0)
Social interaction AND RRBIs	0 (0)	0 (0)	0 (0)	1 (9)	0 (0)	1 (10)	0 (0)
Communication AND RRBIs	0 (0)	0 (0)	0 (0)	1 (9)	2 (15)	1 (10)	7 (11)
Over threshold in all dimensions	0 (0)	0 (0)	0 (0)	2 (18)	5 (38)	7 (70)	52 (85)

Next, we assessed whether the factor structure of autism features was the same across groups. See Table 3 for the outcome of this analysis. Model fit did not decline when fixing loadings across groups, indicating that the three dimensions of autism features clustered similarly in the autistic and SCT groups. Factor loadings [with 95% CIs] for the three dimensions were: Social interaction 0.83 [0.73, 0.92], Communication 0.88 [0.78, 0.97], and RRBIs 0.57 [0.41, 0.73]. The magnitude of the factor loadings indicates that RRBIs were somewhat less central to the autism phenotype, and this was the case equally across groups. However, one parameter did differ between the two groups: the intercept for RRBIs. This indicated that, controlling for Communication and Social interaction scores, the relative level of RRBIs differed ( *r* = -0.27 [-0.45, -0.09], with lower scores in the SCT group).

**Table 3.** Measurement invariance testing of the 3Di-sv across SCT and autistic groups.

Model	Comparison model	*Df* change	*X ^2^* change	*p*-value	CFI	RMSEA [90% CI]
Loadings	No constraints	2	0.67	.717	1.00	.00 [.00, .20]
Intercepts	Loadings	2	7.33	.026	.94	.16 [.00, .30]
Intercepts (3Di-sv communication intercept varies)	Loadings	1	6.60	.010	.93	.19 [.03, .35]
Intercepts (3Di-sv social interaction intercept varies)	Loadings	1	3.98	.046	.97	.12 [.00, .029]
Intercepts (3Di-sv RRBIs intercept varies)	Loadings	1	0.50	.481	1.00	.00 [.00, .17]
Residuals (3Di-sv RRBIs intercept varies)	Intercepts (3Di-sv RRBIs intercept varies)	3	1.94	.585	1.00	.00 [.00, .12]

**Figure 4.**
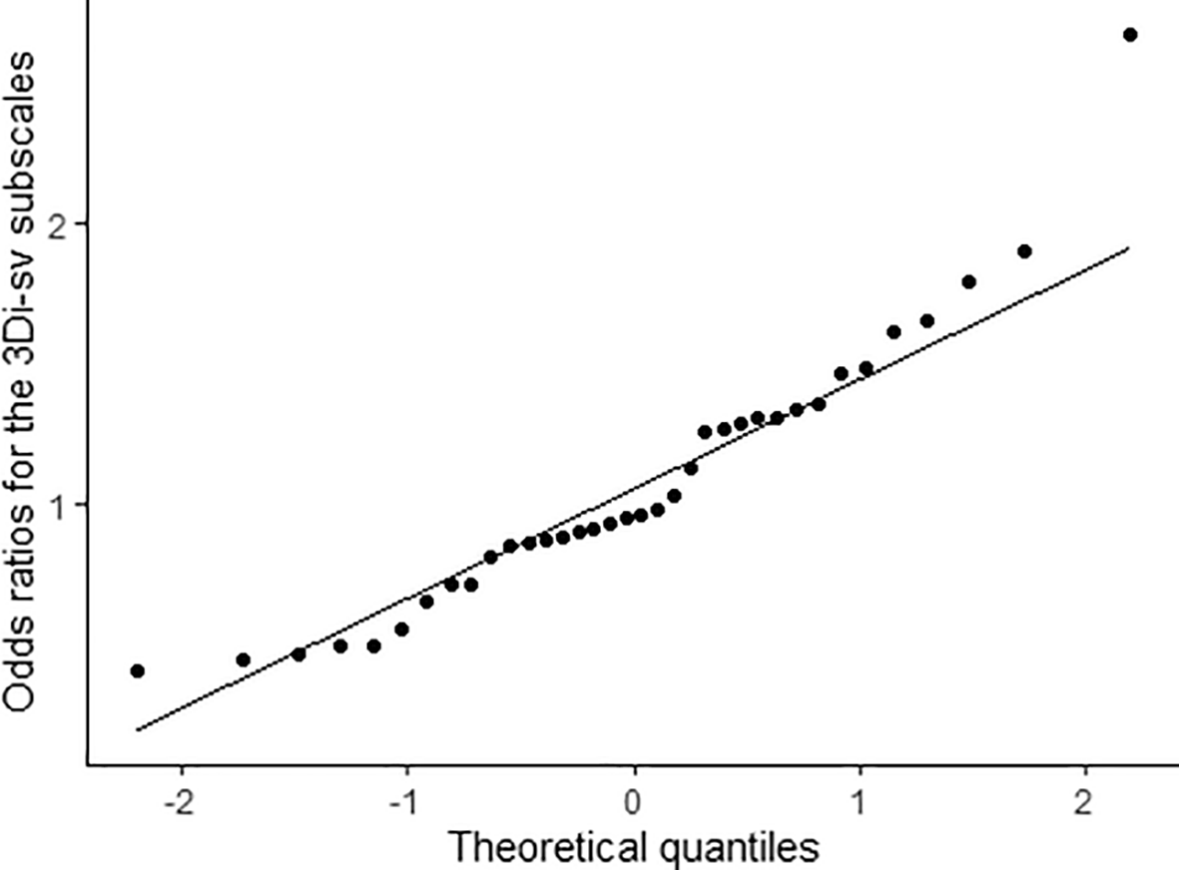
A Q-Q plot showing the distribution of odds ratios for the 3Di-sv subscales. These odds ratios represent the likelihood that young people with a sex chromosome trisomy (SCT) will have an elevated score on a subscale when controlling for scores on the full scale to which the subscale belongs. Generally, the points fall along the line, indicating that the distribution of odds ratios does not differ from a normal distribution and that differences just reflect random noise. There is one outlying point in the top right (for the pronoun errors subscale) which seems to reflect a true difference between groups in presentation of the autism phenotype.

Finally, we took a more fine-grained approach to the 3Di-sv to assess whether groups endorsed the items contributing to the three dimensions differently. We used logistic regression to compute odds that the SCT group would score higher on each subscale when controlling for total scores at the dimension-level. As shown in Table 4, there was little evidence that groups differed in their presentation of the autism phenotype, as odds ratios tended to fluctuate around one as would be expected by chance. In general, these fluctuations were normally distributed as shown in Figure 4, and so attributable to random noise – but there was one outlying exception, the subscale pronoun errors. Young people in the SCT group were disproportionately likely to score higher on this subscale (by 2.66 times, *p* = .002) when controlling for overall autism communication phenotype, compared to young people in the autistic group. We tested whether this was due to greater language difficulties by covarying for language factor scores. This reduced the odds to 1.39, *p* = .446, indicating that language ability influenced group differences in this aspect of the autism phenotype.

**Table 4.** Comparison of subscale responses on the 3Di-sv between SCT and autistic groups. Odds values are shown for the SCT group scoring higher on a subscale compared to the autistic group when controlling for total scores on the dimension to which the subscale belongs. Subscales are on a 2-point scale, and odds represent a change in one point on the scale (i.e. between “never”, “sometimes” and “always”).

Subscale	Odds	Lower 95% CI	Upper 95% CI
*1. Social interaction*			
Direct gaze	0.50	0.18	1.34
Social smiling	1.61	0.72	3.75
Facial expressions for communication	0.47	0.19	1.11
Imaginative play with peers	1.31	0.70	2.56
Interest in children	1.27	0.58	3.04
Response to approach from children	0.87	0.37	2.06
Friendship and play with peers	1.04	0.39	2.73
Sharing interest	1.47	0.61	3.52
Offering to share	0.45	0.22	0.88
Sharing enjoyment	1.36	0.75	2.49
Offering comfort	0.98	0.54	1.79
Using another’s body to communicate	1.26	0.73	2.22
Quality of social overtures	0.90	0.53	1.57
Appropriateness of emotion expressed	1.34	0.73	2.54
Discerning how others are feeling	0.72	0.34	1.50
*2. Communication*			
Pointing to express interest	0.56	0.29	1.07
Use of conventional gestures	1.48	0.68	3.36
Nodding gesture	0.72	0.32	1.60
Shaking head gesture	0.88	0.40	1.87
Social sounds and chat	0.82	0.35	1.90
Reciprocal conversation	0.41	0.14	1.12
Stereotyped or idiosyncratic speech	1.79	0.91	3.71
Inappropriate remarks	1.28	0.64	2.68
Pronoun errors	2.66	1.48	5.16
Neologisms	0.88	0.49	1.56
Spontaneous imitation	0.66	0.39	1.12
Imaginative play	1.13	0.62	2.15
Imaginative play with peers	0.95	0.53	1.72
*3. RRBIs*			
Restricted interests	0.92	0.48	1.79
Unusual preoccupations	0.93	0.46	1.91
Verbal rituals	1.31	0.63	2.83
Non-verbal rituals	1.65	0.77	3.74
Simple mannerisms	0.85	0.41	1.73
Complex mannerisms	0.50	0.20	1.17
Preoccupations with objects	1.90	0.94	4.12
Unusual sensory interests	0.97	0.50	1.91

### Language ability and the autism phenotype across groups

Figure 2 shows raw data on the language measures, and Figure 3 shows scatter plots for the language factor score extracted from these measures against the 3Di-sv dimensions. MANOVAs indicated that the autism phenotype related to language ability measured both through test performance and parent report, with greater language ability predicting fewer autism features on the 3Di-sv dimensions. However, the effect size was relatively small (i.e. language ability explained only 6% of variance in autism features). As shown in Table 5, the group by language ability interactions were non-significant, indicating that the relationship did not differ between groups.

**Table 5.** Nonparametric Multivariate Analyses of Variance (MANOVAs) testing the relationship between language ability and autism features measured by the three 3Di-sv dimensions (social interaction, communication and RRBIs) across the two groups.

	*Df* 1	*Df* 2	*F-*value	*p-*value	Partial *η* ^2^
MANOVA 1					
Trisomy diagnosis	1	89	45.84	<0.001	0.34
Language factor score	1	89	4.78	0.016	0.05
Interaction	1	89	2.35	0.090	0.03
MANOVA 2					
Trisomy diagnosis	1	106	46.21	<0.001	0.30
CCC structural language composite	1	106	6.88	0.004	0.06
Interaction	1	106	1.00	0.342	0.01

### The influence of diagnostic label on level of SEN support

In the third research question, we asked whether there were systematic group differences in the level of SEN provision. The first stage of the regression indicated that level of disability explained 30% of variance in SEN provision, *F* (4, 82) = 10.09, *p* < .001. Addition of group membership in the second stage of regression was significant, *F* (1, 81) = 7.09, *p* = .009, and the full model explained 35% of variance in SEN provision. Controlling for the other variables entered in the regression, a trisomy diagnosis was associated with less SEN provision (0.64 units on the 4-point scale). See Table 6 for full results of the regression analysis.

**Table 6.** Hierarchical multiple regression predicting SEN provision.

	Stage 1		Stage 2	
	*t-*value	*p-*value	*t-*value	*p-*value
Language factor score	-5.69	<.001	-6.47	<.001
Age	-1.86	.066	-1.74	.086
PSC total (psychosocial difficulties)	-0.02	.986	-0.07	.942
3Di-sv factor score (autism features)	2.27	.026	-0.16	.872
Trisomy diagnosis			-2.66	.009

## Discussion

This study investigated the coherence of the autism phenotype: to what extent do autism features necessarily co-occur? And to what extent are autism features necessarily distinct from language? Are these relationships skewed if we simply focus on individuals with an autism diagnosis? Or do we see the same relationships even where a group is selected on the basis of a genetic rather than neurodevelopmental diagnosis? We approached these questions by comparing groups of young people with autism and young people with a sex chromosome trisomy (SCT).

### Similarities between groups

We found no significant difference between the groups in terms of how different aspects of the autism phenotype related to each other. In both groups, social interaction and communication features showed a similar, moderate relationship with the non-social aspects of autism (RRBIs). This replicated previous observations that the social and non-social aspects of the autism phenotype are somewhat dissociable ( Shuster *et al.*, 2014). When modelling autism features in a factor analysis, we found that the factor loading for RRBIs was moderately high (0.57) but rather lower than for the other dimensions (both over 0.80). As noted above, this pattern of factor loadings did not differ across groups.

Language difficulties showed only a weak relationship with the autism phenotype, explaining a small amount of variance (6%) in elevated autism features, and again this did not differ across groups. The weak relationship supports the view that the autism phenotype and language difficulties are relatively distinct, although certain autism-related behaviours may be more common when the individual has a language impairment. As such, the one subscale in the 3Di-sv assessment that was endorsed disproportionately across groups when controlling for total scores was a language-related one – pronoun errors – endorsed more frequently in the SCT group, probably due to the frequency of co-occurring language impairments in this group. However, more generally, language ability was only weakly related to autism features, and this was regardless of group. It was possible that communication difficulties among young people with an SCT may result from language problems, which commonly occur in SCTs ( Bishop *et al.*, 2019) and could mimic autism in this group. However, the lack of much relationship between autism features and language in both groups speaks against this view. Instead, it seems that social communication problems are likely to be more social-cognitive in nature than linguistic. Together these findings support the validity of the autism phenotype. Social difficulties tend to co-occur with RRBIs in autistic individuals; and this is to a similar degree in individuals at genetic risk for developmental problems but who have *not* been selected for autism; and language is a separate dimension of neurodevelopmental disability.

### Differences across groups

However, there were some group differences in presentation of the autism phenotype. The SCT group was much more likely to show clinically-significant features in just some dimensions of the autism phenotype. This indicates that the social aspects of autism can manifest in the relative absence of the non-social aspects, and vice versa. The SCT group also tended to show RRBIs at a lower level compared to individuals in the autistic group with the same level of social interaction and communication features. This difference could be driven by the SCT or autistic group.

On the one hand, presence of an SCT may confer greater likelihood of social difficulties than the non-social aspects of the autism phenotype. However, there are certain caveats to bear in mind here. First, this might predict a dissociation between social and non-social difficulties specifically in the SCT group, but as noted above the factor loadings of different aspects of the autism phenotype did not significantly differ across groups. Also, as shown in Table 2, young people with an SCT who scored above threshold on just some aspects of the autism phenotype were as likely to show RRBIs above the clinical threshold as any other domain. Finally, we should be cautious about any suggestion that the SCT group shows a specific form of the autism phenotype, as there was substantial heterogeneity in presentation (see Figure 3). The exception to this was the 47,XYY group, in which universally high levels of autism features were observed. However, we should be tentative about this finding, as the sample size was small and all individuals in this group were postnatally diagnosed (and so more likely to show a marked phenotype).

If we instead consider whether the autistic group drove the differences, we might suggest that the autistic group “over-selected” for individuals with certain presentations: specifically, with clinically significant difficulties across the board and higher RRBIs than would be expected based on the individual’s social disabilities. Essentially, autism criteria may not map exactly onto the autism phenotype as it manifests “naturally”. In many ways, this is acknowledged in the diagnostic manuals, in which “atypical autism”, “pervasive developmental disorder – not otherwise specified” and “social (pragmatic) communication disorder (SPCD)” have allowed classification of individuals who do not quite meet autism criteria. There has, however, been debate about the utility and validity of these diagnoses (e.g. Norbury, 2014; Walker *et al.*, 2004), and the current study simply underscores the view that diagnostic labels may not carve nature at the seams. Specific questions raised by findings in this study are (a) how to understand and support the difficulties of individuals with an uneven profile of autism features, and (b) how the level of RRBIs should be calibrated against the level of social and communication difficulties in diagnosing autism.

### Practical implications

What are the practical implications of these questions? A key issue is that different diagnostic labels may provide greater or lesser access to resources. Certainly, with regard to research, some neurodevelopmental conditions, e.g. autism, receive much greater research funding and are published on much more extensively than other neurodevelopmental conditions, e.g. dyslexia, dyspraxia and developmental language disorder ( Bishop, 2010). It is less clear what the picture is in the clinical world; certainly, families sometimes express dissatisfaction about the limited resources channelled into diagnostic services and post-diagnostic support ( Crane *et al.*, 2016), although whether this differs by diagnostic label is unclear. In the present study, we found that young people in the SCT group received less special educational needs (SEN) support than young people in the autism group, after controlling for the level of difficulties they experienced (including language disability, autism features, and psychosocial difficulties). There are probably multiple reasons for this. The autism label may have greater currency in educational settings due to widespread knowledge about the condition and the existence of autism-specific services, whereas SCTs are relatively unknown among practitioners. In addition, SCTs may be perceived as rare conditions which require very specific support or conversely that they are genetic “and therefore nothing can be done” (but with this logic, we could say the same about autism, which is currently understood as having a genetic aetiology). If these perceptions are an issue, the advice to practitioners might be to focus not on the SCT label but instead on the difficulties affecting the young person’s day-to-day life, and support the young person with those difficulties using evidence-based interventions.

### Limitations

There are two limitations to bear in mind with this study. First, we used older DSM-IV criteria for autism rather than DSM-5. Autism features were assessed through the 3Di-sv clinical interview, which has been validated for discriminating DSM-IV cases and non-cases. The most important difference between DSM-IV and DSM-5 is the greater representation of sensory features in DSM-5 criteria. Very little is known about sensory reactivity in SCTs, so it is unclear whether this is likely to affect the level of similarity and difference between SCT and autistic groups. We should also note that our assessment was restricted to a single method, i.e. diagnostic interview with an informant, and did not involve direct observation of the young person. The second key limitation is that sample sizes were relatively modest. This limited the analytic methods we could use in the study and the possible subgroup analyses. We ran factor analyses using total scores on the three 3Di-sv dimensions rather than factor-analysing the items within the dimensions, as the latter method would have necessitated a much larger sample size. Nonetheless, we did assess whether families were more or less likely to endorse certain items across groups, and there was little evidence of this.

The other drawback of the small sample size was the limitation on subgroup analyses. It was not possible to run the main factor analysis in separate subgroups defined by trisomy type due to the small numbers. In the present sample, it did seem that the 47,XYY group showed a more severe phenotype than the 47,XXX and 47,XXY groups; however, we cannot be confident in this impression, due to the small numbers and possible confound of timing of diagnosis (as all children in this group were postnatally diagnosed whereas other groups showed a combination of pre and postnatally diagnosed). In addition, it should be noted that existing research does not provide strong reason to expect differences by trisomy type, as studies have typically shown significant variability of symptoms among SCTs but without any clear role to be played by the trisomy type ( Bishop *et al.*, 2019). This variability was also present in the current SCT sample – some children showed very little evidence of autism features whereas others showed elevated levels consistent with a diagnosis of autism (alongside their genetic diagnosis). This was often linked to timing of diagnosis, with postnatally-identified children showing more autism features, as one might expect based on the fact that many of these young people were diagnosed following investigations due to behavioural concerns.

In summary, this study supported the validity of the autism phenotype as comprising related social and non-social domains that were largely distinct from core language ability. However, we showed that the autism phenotype showed subtle differences when comparing a group diagnosed with autism to a group with genetic conditions conferring a heightened likelihood of neurodevelopmental disability. This supports the view that diagnostic criteria may not map exactly onto the autism phenotype as it manifests “naturally”, which may result in some individuals falling through the diagnostic cracks. Given the link we found between diagnostic label and the level of special educational needs provision, this may impact the support an individual can access.

## Data availability

Open Science Framework. Data and scripts: children in clinical groups. DOI: https://doi.org/10.17605/OSF.IO/7WDVU ( Wilson & Bishop, 2022b).

This project contains the following underlying data:

- PARENT SURVEY
- **CCC.csv** Children's Communication Checklist ( Bishop, 1998), a parent-report questionnaire assessing structural and pragmatic aspects of language, as well as autistic features. File presents item-level responses to all items; then number of unscored items for the structural, pragmatic, social and interests subscales; then total scores on each subtest. Total raw scores and prorated total scores are given (where parents left up to 20% of items unscored for a subscale, scores were prorated by the mean score for the rest of the items in that subscale; where more than 20% of items were unscored, that subscale was recorded as invalid). Item-level responses are coded as follows: 0=does not apply; 1=applies somewhat; 2=definitely applies; NA=don't know/can't judge. Note that item-level scores have been reverse coded for positively-worded items.
- **PSC17.csv** Pediatric Symptom Checklist, a parent-report questionnaire of general psychosocial impairment ( https://www.massgeneral.org/psychiatry/treatments-and-services/pediatric-symptom-checklist). In addition to total scores, factor analysis supported the extraction of subscores for attention (5 items), internalising (5 items) and externalising difficulties (7 items; Gardner *et al.*, 1999). For each of these subscales, the file presents item-level responses followed by two summary scores: subscale total and positive screen for impairment on that subscale. At the end of the file, total scores for the whole scale, positive screen for impairment on the whole scale, and number of missing items are shown. Where any items are left unscored for a subscale, total score for that subscale is recorded as invalid for that participant; for the categoric variables (impaired or not impaired), scores are provided if the participant would necessarily have screened positively or negatively irrespective of how the missing items were answered. Where three or fewer items are left unscored on the whole scale, total scores are calculated ignoring those unscored items; the whole scale is recorded as invalid if a participant leaves more than three items unanswered. Item-level responses as coded as follows: 0=never; 1=sometimes; 2=often; NA=unanswered.
- **participant.information.csv** File presents data for each child in the following order: their study ID; gender; age at the time the parent completed the survey; the UK school year group they belong to based on their birthday; whether they completed most/all of the test battery (1=most/all tests completed; 0=one or fewer tests completed); whether they have an autism diagnosis; whether they have a diagnosis of a sex chromosome trisomy (SCT); whether an SCT diagnosis was prenatal (0=postnatal; 1=prenatal; NA=no trisomy diagnosis); the type of trisomy; whether they have been diagnosed with a language or literacy disability (e.g. SLI, DLD, dyslexia, etc.); and their level of SEN (special educational needs) support (0=none; 1=some low-intensity support in mainstream school; 2=high intensity support in mainstream school; 3=attends special school; NA=home-schooled).
- PARENT INTERVIEW
- **3di.csv** The developmental, diagnostic and dimensional (3Di) assessment was administered over the phone ( Santosh *et al.*, 2009). The file presents scores on the Social Interaction, Communication, and Repetitive and Restrictive Behaviour and Interests (RRBIs) subscales (the Nonverbal Communication score is based on a subset of the Communication items). Autism case-ness is shown in the final column; 3Di criteria for autism are a Social Interaction score of 10 or over, plus a Communication score of 8 or over and/or an RRBI score of 3 or above.
- TEST BATTERY (See Wilson and Bishop (2022a) for normative data and psychometric analysis of the test battery in almost 400 non-autistic children. Please note that a small number of items were dropped from the tests based on this psychometric analysis, and data for these items are not given in the files uploaded here. In addition, z-scores have been computed for some of the tests, and are included in files with "z-score conversion" in the title.)
- **Grammar.csv** Receptive Grammar task, in which participants listen to sentences and decide if they are grammatical. They hear the following instructions: “Some of the sentences will sound good, but some of the sentences will sound bad. There might be a missing word. Or the wrong word might be used. Or the order of the words might be weird. If the sentence is good, click the green tick. If the sentence is bad, click the red cross”. There are 50 items: in 4 sentences the words are in a random order and should be easily rejected, 20 items are taken from McDonald (2006) and showed high accuracy in primary school children, and 26 items are a subset of our adult version of this test ( Wilson *et al.*, 2019); these latter items were chosen on the basis of high accuracy and high item-total correlations. Excluding the 4 randomly ordered sentences, 23 items do not follow typical syntax or use incorrect word forms (e.g. incorrect tenses) and 23 follow typical English grammar. Examples of incorrect items include: “The teacher told the story the children” and “I went out after I have eaten dinner”. There was one measured variable: the sum of items currently answered (out of 50). The file presents item-level accuracy and total scores.
- **Vocab.csv** Receptive Vocabulary task, in which participants choose which of four pictures is related to a word. Participants hear a sequence of 39 words and for each word, they are presented with four pictures on the screen. They are asked to “chose which picture goes best with the word”. The words include nouns, verbs and adjectives, and vary in approximate age of acquisition from 5 to 12, with similar numbers of easy and harder words; two experienced teachers independently rated the ages at which they would expect 50% and 90% of children in a typical class to be familiar with the word. There was one measured variable: the sum of items correctly answered (out of 39). The file presents item-level accuracy and total scores.
- **Implicature.csv** Implicature Comprehension Test, in which participants watch a series of cartoon videos, in each of which two characters produce a short utterance one after the other. Together the utterances form a conversational adjacency pair; in most cases, this is a question and answer. After this dialogue, participants hear a comprehension question, and they give a yes-no-don’t know response by clicking buttons on the screen. For 33 items, participants need to process implied meaning to answer the question, as the second character provides an indirect response to the first character. An example item includes: Character 1: “Could you hear what the police said?” Character 2: “There were lots of trains going past.” Comprehension Question: “Do you think she heard what the police said?” Correct Answer: “No.” There are also 10 items where the answer is more explicit; these serve as positive control items. An example item includes: Character 1: “Did you see the policemen earlier on?” Character 2: “I saw them standing on the platform.” Comprehension Question: “Do you think he saw the policemen?” Correct Answer: “Yes.” From these items, there were two measured variables: sum of implicature items correctly answered (out of 33) and sum of explicit-response control items correctly answered (out of 10). The file presents item-level accuracy and total scores.
- **Inference.csv** Children's Test of Local Textual Inference, in which participants hear two brief sections of a short story (about 90 words per part). After each section, they hear ten questions and four possible answers for each one. “We don’t know” is an answer option for every question, and is the correct answer to four questions. Participants click the correct option on the screen. As well as auditory presentation of all materials, everything is shown in text-based form on the screen. Participants are informed at the start that the short story sections will remain on the screen while they are answering questions about that section. Participants need to make inferences based on the short story to answer the questions. The short story starts as follows: “Unfortunately, the family couldn’t go swimming. The sea was rougher and colder than expected. Instead, Billy spent the whole morning playing a ballgame with his sister, Susie.” An example question is: “What had Billy planned to do?” Participants chose their answer from the following options: “play a ballgame”, “go swimming”, “walk along the sea”, and “we don’t know”. There was one measured variable: the sum of items correctly answered (out of 20). The file presents item-level accuracy and total scores.
- **Overtures.csv** Social Overtures task, in which participants hear 23 utterances spoken by a character to a conversational partner. Eleven are social overtures that attempt to engage the partner in a conversation (e.g. “I can’t believe what happened today.”) and twelve are not conversational bids (e.g. “I’m going to have a shower now.”). Participants listen to instructions explaining that “There are different reasons why we say things to other people. Sometimes, we want to start a conversation. We want the other person to ask us questions and say lots of things to us. Other times we just want to tell the other person something very quickly. We don’t always want to start a long conversation.” They are then asked for each sentence whether the speaker wants a conversation or not, and to indicate their answer by clicking yes-no buttons. There was one measured variable: the sum of items correctly identified as a social overture or not (out of 23). The file presents item-level accuracy and total scores.
- **Matrices.csv** Animal Matrices nonverbal reasoning task, in which participants are presented with a sequence of 16 2×2 matrices on the computer screen. In three of the boxes of each matrix, there are cartoon pictures of animals, and the fourth box is empty. The animals in the three boxes vary along six dimensions: species, colour, size, number, direction faced, and position in the box. There are systematic relationships between the three animals, and participants need to deduce which of five options fits in the empty box. For example, the top two boxes may show red lions, one big and one small, and the bottom left box may show a big yellow horse; the correct option to fill the empty box would be a small yellow horse. There was one measured variable: the sum of items correctly answered (out of 16). The file includes item-level accuracy and total scores.

Data are available under the terms of the Creative Commons Attribution 4.0 International license (CC-BY 4.0).
