# Supplementary material for: Does the autism phenotype differ when selecting groups by neurodevelopmental versus genetic diagnosis? An observational study comparing autism and sex chromosome trisomy
Source: F1000Res. 2022 May 25;11:571. [Version 1] doi: 10.12688/f1000research.121878.1 (PMC9758443; doi:10.12688/f1000research.121878.1)
Supplement: Peer review history

# Peer review: Reviewer response for version 1

**DOI:** 10.5256/f1000research.133788.r154704

**Published:** 2022-12-16

**Authors:** Nancy Raitano Lee

**Affiliations:** 1 Department of Psychological and Brain Sciences, Drexel University, Philadelphia, PA, USA

Thank you for the opportunity to review “Does the autism phenotype differ when selecting groups by neurodevelopmental versus genetic diagnosis? An observational study comparing autism and sex chromosome trisomy” for *F1000 Research*. Wilson and Bishop pose a compelling question about the validity of the assumptions that underlie the autism diagnostic criteria (i.e., that social and non-social features of autism cluster together but are distinct from structural [i.e., non-social] language skills). They utilize a clever design by comparing associations among the triad (using DSM-IV as opposed to DSM-5 terminology) of autism symptoms and structural language skills in two samples – one defined by genotype (sex chromosome trisomies [SCTs]; n= 49) and the other by phenotype (those with behaviorally-ascertained autism diagnoses; n = 61). The authors report that autistic symptoms cluster together similarly in the behaviorally-defined autism group and the genetically defined SCT group. They also note that language skills do appear to be distinct from autism symptomatology in the two groups. However, they note two characteristics of the autism symptom presentation in youth with SCT that differ from those with behaviorally-ascertained autism: (a) those with SCT were more likely to show clinically significant elevations in some but not all ASD symptom clusters, and (b) those with SCT had lower levels of repetitive behaviors/restricted interests (i.e., non-social features of ASD).

 Overall, the research questions were clearly stated, the study was well-designed, and the research methods/statistical analyses employed were rigorous. In what follows, I note some questions I had that may be helpful for the authors to consider.

  

1. Although I understand the use of the term ‘naturally’ when referring to the clustering of ASD features among youth with SCTs as opposed to behaviorally-defined ASD, I struggled a bit with the use of this terminology. Specifically, I was concerned that the reader who is naïve to autism research may wonder if this implies that behaviorally-defined ASD has less of a biological origin than ASD/ ASD features in those with SCTs (as ‘natural’ could imply that one group has a biologically driven symptom presentation and the other group does not).  Is there another way to describe this distinction?
2. I had a question about how exclusion criterion 3 (exclusion of those who were nonverbal or had phrase-level speech) may have impacted the pattern of findings. Upon reviewing the tests used to assess language, I understand why the authors imposed this exclusion to participation. However, I wonder if this influenced the findings. For example, had the authors permitted the inclusion of those with more limited language abilities, might you have seen less dissociability between language skills and social communication skills? By constraining structural language skills in the sample, could this have influenced associations observed between language skills and autism symptomatology. I don’t know that I have the answer, but this is certainly something I wondered as I read this paper and carefully considered the results.
3. When discussing study findings, it may be beneficial to reference the literature on autism symptomatology in other neurogenetic disorders at heightened risk for ASD (e.g., fragile X syndrome, Down syndrome, Williams syndrome, Smith-Magenis syndrome). This could lead to an even richer discussion of the findings. Although studies of ASD symptomatology in these other neurogenetic syndromes may not have set out to evaluate the validity of the autism behavioral phenotype, per se, they are likely to speak to issues related to how  autism symptoms cluster together in other neurogenetic syndromes as well as differences in symptom severity in the social and non-social aspects of ASD.  

Again, thank you for the opportunity to review this very interesting manuscript!

Is the work clearly and accurately presented and does it cite the current literature?

Yes

If applicable, is the statistical analysis and its interpretation appropriate?

Yes

Are all the source data underlying the results available to ensure full reproducibility?

Yes

Is the study design appropriate and is the work technically sound?

Yes

Are the conclusions drawn adequately supported by the results?

Yes

Are sufficient details of methods and analysis provided to allow replication by others?

Yes

Reviewer Expertise:

language, executive function, and social development among youth with neurogenetic syndromes

I confirm that I have read this submission and believe that I have an appropriate level of expertise to confirm that it is of an acceptable scientific standard.

# Peer review: Reviewer response for version 1

**DOI:** 10.5256/f1000research.133788.r145590

**Published:** 2022-08-08

**Authors:** Lu Qiao

**Affiliations:** 1 Department of Pediatrics, Columbia University Medical Center, New York, NY, USA

In the paper titled “Does the autism phenotype differ when selecting groups by neurodevelopmental versus genetic diagnosis? An observational study comparing autism and sex chromosome trisomy”, Wilson et al. analyzed 61 young people diagnosed with autism and 49 with sex chromosome trisomy for whom the psychometric measures including autism phenotype, language ability, general psychosocial impairment and special educational needs support were available. The authors investigated the difference of autism phenotypes, different relationships of language ability with autism features and difference in the amount of special educational needs across above samples defined by behavioural versus genetic diagnoses. No significant difference of autism phenotypes between groups and autism features were distinct from language ability in both groups. Controlling for level of difficulties, SCT group received less special educational needs than autism group. This study supports that autism diagnostic criteria may not map exactly onto the autism phenotype.

 In their paper, the authors did an excellent job controlling for prenatal or postnatal samples, age or school year, levels of difficulties, etc in the analysis. The neurodevelopmental abilities or autism features are highly correlated with age or school year, levels of difficulties or even diagnosed phrase, which are stated in the introduction. They detect the proportion, variances and covariances instead of mean score. They removed the effect of age on the language factor using linear regression or take age and language ability as independent variables. I believe these are very reasonable methods to solve the questions. One concern is small sample size in the multi-group confirmatory factor analysis. The other things that I liked about this paper are organized logically in the introduction and discussion part. The paper is generally well written and structured.

 Below I have provided numerous remarks on the text or figures:

1. In the introduction part, adding more details in the relationship between SCT and autism especially in genetic and phenotype aspect would be constructive. I thought this may help us readers understand the purpose at the beginning of the paper.
2. In figure 1, is the model B showing the autism in general population? Is it more helpful to highlight autism in red to explain model B? What’s the reference of those two models?
3. In figure 3, can you show the scores on x and y axis to clarify the measure values?

Is the work clearly and accurately presented and does it cite the current literature?

Yes

If applicable, is the statistical analysis and its interpretation appropriate?

Yes

Are all the source data underlying the results available to ensure full reproducibility?

Partly

Is the study design appropriate and is the work technically sound?

Yes

Are the conclusions drawn adequately supported by the results?

Yes

Are sufficient details of methods and analysis provided to allow replication by others?

Yes

Reviewer Expertise:

rare disease, genetic variants, genome-wide association study, multi-omics study and neurodevelopmental outcomes

I confirm that I have read this submission and believe that I have an appropriate level of expertise to confirm that it is of an acceptable scientific standard.
